# Risk Assessment of Micropollutants for Human and Environmental Health: Alignment with the Urban Wastewater Treatment Directive in Southeastern Spain

**DOI:** 10.3390/toxics13040275

**Published:** 2025-04-04

**Authors:** Lissette Díaz-Gamboa, Agustín Lahora, Sofía Martínez-López, Luis Miguel Ayuso-García, Isabel Martínez-Alcalá

**Affiliations:** 1 Social Responsibility, Sustainability and Innovation Group (GAIA), Universidad Católica de Murcia (UCAM), Av. de los Jerónimos, 135, 30107 Murcia, Spain; pldiaz@ucam.edu; 2 Regional Entity for Sanitation and Wastewater Treatment in the Region of Murcia (ESAMUR), C. Santiago Navarro, 4, 30100 Murcia, Spain; agustin.lahora@esamur.com; 3 Environmental Department, National Technological Centre for the Food and Canning Industry (CTNC), C. Concordia, S/N, 30500 Murcia, Spain; sofiamartinez@ctnc.es (S.M.-L.); ayuso@ctnc.eu (L.M.A.-G.)

**Keywords:** emerging contaminants, human risk, ecotoxicological risk, regenerated water, UWWTD

## Abstract

The reuse of reclaimed water is essential for sustainable water management in arid regions. However, despite advancements in Wastewater Treatment Plants (WWTPs), certain micropollutants may persist. To address these challenges, the recently enacted European Urban Wastewater Treatment Directive (UWWTD) has established strict standards focused on monitoring twelve specific indicator compounds. In line with this, the present study aims to evaluate the concentrations and potential risks of these twelve UWWTD-designated compounds across various water sources, including surface water, groundwater, and effluents from a WWTP in the southeast of Spain. Although none of the evaluated water sources are, as expected, intended for human consumption, risks were assessed based on worst-case scenarios that could amplify their impact. The study assessed potential risks to human health across different age groups and ecosystems, focusing on key organisms such as fish, daphnia, and algae, using empirical assessment approaches. The risk assessment identified a low risk for most compounds regarding human health, except for citalopram (HRQ = 19.116) and irbesartan (HRQ = 1.104), which showed high human risk quotients (HQR > 1) in babies, particularly in reclaimed water. In terms of ecotoxicological risk, irbesartan presented the highest ecological risk quotient (ERQ = 3.500) in fish, followed by clarithromycin, with algae (ERQ = 1.500) being the most vulnerable organism. Furthermore, compounds like citalopram, venlafaxine, and benzotriazole exhibited moderate ecological risks (ERQ between 0.1 and 1) in the reclaimed water, and their risk was reduced in surface water and groundwater. Finally, this study discussed the potential impacts of elevated concentrations of these emerging compounds, emphasizing the need for rigorous wastewater monitoring to protect human health and ecosystem integrity. It also revealed notable differences in risk assessment outcomes when comparing two distinct evaluation approaches, further highlighting the complexities of accurately assessing these risks.

## 1. Introduction

In recent decades, the reuse of reclaimed water has emerged as a critical strategy for sustainable water management, particularly in arid and semi-arid regions, such as the southeastern region of Spain [1]. This practice is especially pertinent in areas facing acute water shortages and allows us to optimize water resources by recycling wastewater for purposes such as irrigation, maintaining the ecological flow of rivers and aquifer recharge. However, the discharge and use of reclaimed water for these applications raises significant concerns regarding the presence and impact of emerging compounds in the environment [2]. Despite the efficiency of treatment processes, certain compounds may persist or undergo partial degradation, leading to the presence of micropollutants in reclaimed water [3]. These compounds encompass a broad range of substances, including pharmaceuticals, personal care products, industrial chemicals, among others, which may persist through the water cycle and accumulate in various environmental compartments such as soil, water bodies, and even biota. Their increasing detection in different environmental matrices has heightened concerns about their potential impacts on human health and ecological systems [4].

This has driven the need for supplementary treatment methods and stricter regulations. In response, Directive 91/271/EEC was revised in 2024, now known as the Urban Wastewater Treatment Directive (UWWTD), to align with major European environmental strategies such as the European Green Deal, the Zero Pollution Action Plan, and the Circular Economy Plan [5,6]. The updated directive establishes stringent requirements, including an 80% reduction in micropollutants, along with the mandatory implementation of quaternary treatments for plants with capacities exceeding 150,000 h-e and facilities over 10,000 h-e in regions where micropollutants pose potential risks to human health and the environment. To comply with the directive, systematic monitoring of key micropollutants is essential to confirm the effectiveness of treatment processes in reducing these contaminants. The directive focuses on tracking twelve indicator substances, which are grouped for better management. Category 1 includes eight pharmaceuticals considered “substances that can be very easily treated” indicating their efficient removal through standard wastewater treatment. Category 2 encompasses four substances that are also “substances that can be easily disposed of” though ongoing attention is required due to their increasing presence in wastewater systems [6].

Previous research has documented the significant effects that emerging contaminants can have on human health and environmental integrity. In humans, these compounds are associated with severe health impacts such as endocrine disruption, the development of antibiotic resistance, and neurotoxicity, underscoring their profound implications for public health [7]. For example, the presence of antimicrobial drugs in the aquatic environment fosters the development of multi-drug-resistant bacteria, such as *E. coli*, a Gram-negative bacterium that exhibits increased resistance to sulfonamides when exposed to wastewater effluents [8]. Vulnerable groups, including children, pregnant women, and the elderly, may be more sensitive to even low levels of these contaminants, especially pharmaceuticals, due to their more delicate physiological conditions [9].

At the same time, these substances have been shown to significantly impact ecosystems. Exposure to certain pharmaceuticals can disrupt hormonal levels in aquatic organisms such as fish and amphibians, leading to negative effects on reproduction and survival [10]. Organisms like daphnids and algae, which play crucial roles in aquatic ecosystems, are often used to assess the ecological consequences of pollutants. Daphnids, important herbivores and prey, may experience reduced reproduction and increased mortality when exposed to emerging contaminants, thus disrupting food webs [11]. Algae, as primary producers, can show inhibited growth and photosynthesis when exposed to these substances, affecting oxygen production and nutrient cycling, leading to broader ecosystem effects [12]. Recent studies further highlight the dangers of emerging contaminants. For instance, a study in Colombia reported high concentrations of acetaminophen in urban wastewater, exceeding established limits in this region [13]. These concentrations surpassed those defined by [14], which determined that 10 µg/L of acetaminophen was sufficient to induce teratogenic, neurotoxic, and cardiotoxic effects in the embryos and larvae of *Clarias gariepinus*. Similarly, the presence of ibuprofen in aquatic environments has been associated with disruptions in the reproductive patterns of the Japanese medaka (*Oryzias latipes*), interfering with hormonal regulation and leading to altered spawning behaviors and reduced fertility [15].

Although these compounds, along with many others, have been extensively studied and their ecological and human health risks assessed, there is still limited information available regarding a wide range of commonly used pharmaceuticals, as well as a lack of data on non-pharmaceutical contaminants. This knowledge gap remains a growing concern. Therefore, there is an urgent need for continued research on emerging pollutants and their associated ecological and human health risks, particularly those listed for observation or regulated under current frameworks, to fully understand their potential dangers. Moreover, in the context of arid regions, such as some areas of Spain, it is especially important to keep these types of studies up to date. These regions frequently engage in water reuse practices, and treated wastewater is often used for high-care applications such as agricultural irrigation. Given the potential risks posed by emerging pollutants, it is crucial to ensure that the safety of such water use is continuously evaluated and that regulations are adapted accordingly.

Despite their widespread presence, the impact of emerging compounds on specific age groups and reference organisms regarding human and ecological health remains poorly understood. However, there are current methodologies for the evaluation of both human and ecological risks. Human risk is assessed through Drinking Water Equivalent Levels (DWEL), which are tailored to different age groups. These levels are calculated based on the maximum amount of a contaminant in drinking water that is not expected to cause adverse effects on human health, considering daily intake and other physiological and metabolic factors specific to each age group [16,17]. For environmental risk assessments, Predicted No Effect Concentrations (PNEC), derived from toxicity tests conducted on model organisms representing different trophic levels, are used to provide insights into the potential impacts on aquatic ecosystems. PNECs provide an estimation of the concentration of a chemical compound in the environment that should not cause adverse effects on any species within the ecosystem [16,18,19]. One of the main challenges in risk assessment of emerging compounds is the lack of complete and accurate data on their toxicity and behavior in the environment. Many emerging compounds have not been extensively studied, and their long-term effects on human health and ecosystems are unknown. Variability in the sensitivity of different organisms and the interaction between multiple contaminants further complicate accurate risk assessment [17,19]. Given the growing recognition of the risks posed by emerging compounds, it is crucial to investigate their presence and distribution in environmental samples, particularly those influenced by reclaimed water from WWTPs.

Based on this context, this study aims to assess the potential risks to human health and local ecology posed by certain emerging contaminants, analyzing their possible effects in different water matrices. We examine the occurrence, concentration, and distribution of twelve indicators established in the UWWTD in diverse water samples—surface water, groundwater, and reclaimed water in the Murcia Region, Spain. Importantly, it should be noted that neither the surface water nor the groundwater in this study are within catchment areas designated for water intended for human consumption, which mitigates some of the potential health risks. Using an integrated approach, we evaluate the risks of human exposure across different age groups and the ecological risk for indicator organisms such as fish, daphnia, and algae, highlighting the potential effects of contamination. This analysis is crucial for developing effective strategies to mitigate the risks associated with these micropollutants and for informing regulatory policies aimed at protecting public health and environmental integrity. Our study specifically contributes to understanding the risks posed by these emerging contaminants in the Murcia Region, an area characterized by arid and semi-arid conditions where water reuse is a key strategy for addressing water scarcity. Given that reclaimed water is widely used for various purposes, including agricultural irrigation in this area, continuous monitoring and risk assessment are essential to prevent potential adverse effects on both ecosystems and human health.

## 2. Materials and Methods

### 2.1. Sampling Sites

**Figure 1:**
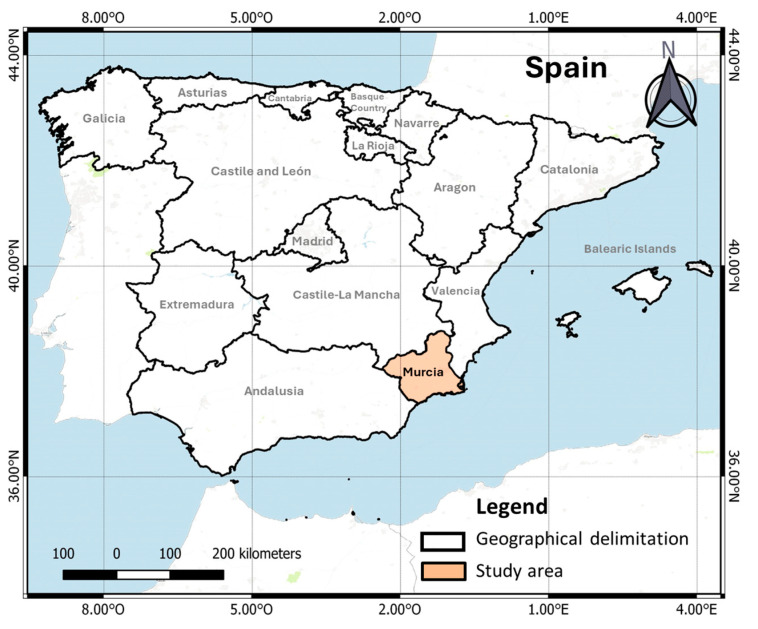
Location of the study site in the southeast of Spain, in the Murcia Region.

The study site is located in the Region of Murcia, Spain (Figure 1). Sampling points in this study encompass surface water samples, groundwater samples, and reclaimed water directly sourced from the effluent of the local WWTP. These sampling sites were selected due to their exposure to treated effluents from WWTPs and other anthropogenic activities. Consequently, they are expected to contain different levels and types of emerging contaminants. The WWTP is a facility with a treatment capacity of 12,775,000 m^3^ per year. It serves a population of 176,921 inhabitants and utilizes a secondary treatment system based on conventional activated sludge, followed by an additional treatment in storage lagoons.

### 2.2. Collection of Samples

In this study, surface water, groundwater, and WWTP effluent samples (n ≥ 6) were collected, processed, and analyzed according to UNE-EN ISO/IEC 17025 standards [20]. The sampling period extended from April 2022 to November 2023, providing a broad temporal scope and including possible variations in water quality. During this period, spot sampling techniques were employed to collect samples at specific intervals, offering snapshots of water quality at distinct points in time. This approach was essential for detecting transient changes and short-term events that could impact water quality. To ensure sample integrity, pre-rinsed amber glass bottles of 1 L were used for collection.

### 2.3. Micropollutants Analysis

The list of target compounds included in the present study comprises substances with diverse applications and chemical characteristics, commonly detected in water, such as pharmaceuticals and industrial chemicals. These compounds are known for their widespread consumption and potential negative effects. The classification used to categorize the studied compounds corresponds to two categories (Category 1 and Category 2) established by the UWWTD, which is based on treatment and removal processes. These compounds of emerging concern were analyzed according to the German standard methods specified in DIN 38407-47:2017-07 [21] for water and wastewater examination. The analysis employed high-performance liquid chromatography (HPLC) coupled with mass spectrometric detection, utilizing either tandem mass spectrometry (MS/MS) or high-resolution mass spectrometry (HRMS) (SCIEX, Framingham, MA, USA). The samples underwent direct injection prior to analysis to ensure the integrity of the samples and minimize procedural errors.

### 2.4. Estimation of Human and Ecological Risk Quotients

While the water samples in this study are not intended for direct consumption by the population, they are used for various purposes including agriculture. Therefore, it is crucial in this scenario to ensure that there is no risk to human health associated with potential direct consumption of water or crops containing the analyzed emerging compounds. To assess the contaminants posing the highest human and/or ecological risks in these water samples (surface water, groundwater, and reclaimed water), two risk quotient approaches (Approaches A and B) were considered, both based on the concentrations identified in the detection analyses. However, the primary risk assessment was conducted using approach A, while approach B was used for comparison. Compounds with values below the LOQ in all measurements were excluded from the risk assessment, as these values do not provide accurate quantifiable data for the calculations.

#### 2.4.1. Human Risk Quotient

The primary methodology for the calculation (Approach A) involved determining the age-specific human risk quotient for each emerging compound using a method adapted from [17]. This involved dividing the maximum concentration found in the environmental sample (MEC) by the respective age-specific Drinking Water Equivalent Level (DWEL) as shown in Equation (1).

(1) $${HRQ}_{A}=\frac{MEC}{DWEL}$$

Considerations for DWELs include the acceptable daily intake or the risk-specific dose for both non-cancerous and cancer-causing effects of individual compounds, average body weight in kilograms, and average daily water consumption across various age categories. Additionally, factors such as absorption rate in the gut and regularity of contact with the substance are considered [22]. In this study, HRQ was calculated based on these DWEL computations for babies, teenagers, and adults (Table 1). ADI values were referenced across various scientific publications. When multiple values were available, the lowest was chosen to ensure maximum safety. To model a conservative “worst-case” scenario, the maximum measured concentrations were applied. According to Yang et al. (2017) [23], an HRQ_A_ value greater than 1 may indicate a possible health hazard for humans. A value ranging from 0.1 to 1 warrants a more thorough investigation, while an HRQ value below 0.1 is generally not associated with significant health risks.

#### 2.4.2. Ecological Risk Quotient

Following the same approach A, the ecological risk quotient (ERQ_A_) was calculated by dividing the measured environmental concentration (MEC) of each compound by the derived PNEC value, as shown in Equation (2) [17,30].

(2) $${ERQ}_{A}=\frac{MEC}{PNEC}$$

The Predicted No-Effect Concentration (PNEC) was calculated following the principles outlined by [30] (Table 1). The PNECs were derived from acute and chronic toxicity data for representative aquatic species, using assessment factors in accordance with the ECHA guidance. PNEC values used in this study were primarily sourced from the United States Environmental Protection Agency (EPA) website, which provides standardized and peer-reviewed values for a range of chemical compounds related to their ecotoxicity. For compounds lacking direct PNEC values from the EPA, estimates based on experimental data or extrapolations from recent studies were used [17,24,25,26,27,28]. In cases where PNEC values or their derivatives were not available for certain compounds, such as irbesartan and methylbenzotriazole, no values have been established or reported in the available literature. While this data gap introduces some uncertainty, it is not expected to significantly affect the overall ecological risk assessment, as the available PNEC values provided a solid foundation for the evaluation.

The lowest and most current possible PNEC value was used to be protective and to assess the “worst-case scenario” in each case. The ERQ was determined by considering the PNECs for various trophic categories, including fish, daphnia, and algae. These calculations involved incorporating the EC_50_ (the concentration causing a 50% reduction in specific biological activity) or the LC_50_ (the concentration at which 50% of test organisms die) along with an assessment factor (AF 1000), designed to address the conservative nature of the approach and associated uncertainties (Equation (3)) [17]. The PNECs applied in this analysis are showed in Table 1.

(3) $$PNEC=\frac{{EC}_{50}\;or\;{LC}_{50}}{AF}$$

ERQ_A_ is classified into three categories. A value above 1 indicates a potential risk to the local ecology, while a moderate ecological risk falls between 0.1 and 1. A value below 0.1 denotes a low ecological risk [17,31]. This methodology accounts for the Risk Quotients of individual compounds; however, this study acknowledges that substances typically exist in the environment as mixtures rather than in isolation from an ecological viewpoint.

#### 2.4.3. Alternative Approach for Human and Ecological Risk Assessment

As a comparative analysis, this study applied an alternative methodological approach for assessing human and ecological risk (Approach B), based on the mathematical framework proposed by Munné et al. (2023) [32]. Originally designed for drinking water, this methodology was adapted to evaluate environmental waters not intended for direct human consumption, offering an alternative perspective on potential risks to both humans and aquatic organisms. Munné et al. (2023) [32] established guideline values (GVs) for pharmaceutical and non-pharmaceutical compounds using Australian guidelines (NRMMC-EPHC-NHMRC, 2008) [33] to derive threshold values for human health risk assessment. The GV is calculated as follows (Equation (4)):

(4) $$GV=\frac{ADI\times bw\times P}{V},$$

where ADI is the acceptable daily intake of the compound (Table 1), bw is the body weight based on age group values from Sharma et al. (2019) [17], P is the proportion of total intake attributed to water consumption (10% or 20%), and V is the daily water intake (2 L). Once the GV is determined, the human risk quotient (RQ) is calculated as the ratio of the MEC to its corresponding GV (Equation (5)).

(5) $${HRQ}_{B}=\frac{MEC}{GV}$$

This alternative approach was applied to the 12 compounds regulated under the UWWTD. Although this study focuses on environmental waters, including reclaimed, surface, and groundwater that are not designated for human consumption, this methodological comparison provides insight into potential risks under hypothetical exposure scenarios.

For the ecotoxicological risk assessment, the approach proposed by [32] employs PNEC as the GV. Ecotoxicological risk (ERQ_B_) is then determined using Equation (2), with PNEC values sourced from Table 1, which are the same as those used in the initial approach. In this estimation (Approach B), HRQ_B_ and ERQ_B_ are considered simple threshold measures, where values greater than 1 indicate a potential risk, while values lower than 1 suggest minimal concern.

### 2.5. Statistical Analysis and Data Treatment

Statistical analyses were conducted to compare both micropollutant concentrations and risk values (HRQ and ERQ) across reclaimed water (effluent WWTP), surface water, and groundwater. Since most of the data did not follow a normal distribution, the Kruskal–Wallis one-way ANOVA test was applied (*p* < 0.05) to determine significant differences among the three groups. Dunn’s post hoc test was then used for multiple comparisons (*p* < 0.05, *p* < 0.01 and *p* < 0.1) to identify which specific groups differed significantly. Given the high variability in the data, a significance threshold of *p* < 0.1 was also considered to detect potential trends that might not be evident under more stringent criteria. To better visualize the distribution of HRQ and ERQ data, box plots (whisker plots) were generated, illustrating the median, interquartile range (P25–P75), and the minimum and maximum values within the non-outlier range. All descriptive statistics were calculated using Excel, while additional statistical analyses and graphing were performed in GraphPad Prism 9.4.1.

## 3. Results and Discussion

### 3.1. Occurrence of Micropollutants

**Figure 2:**
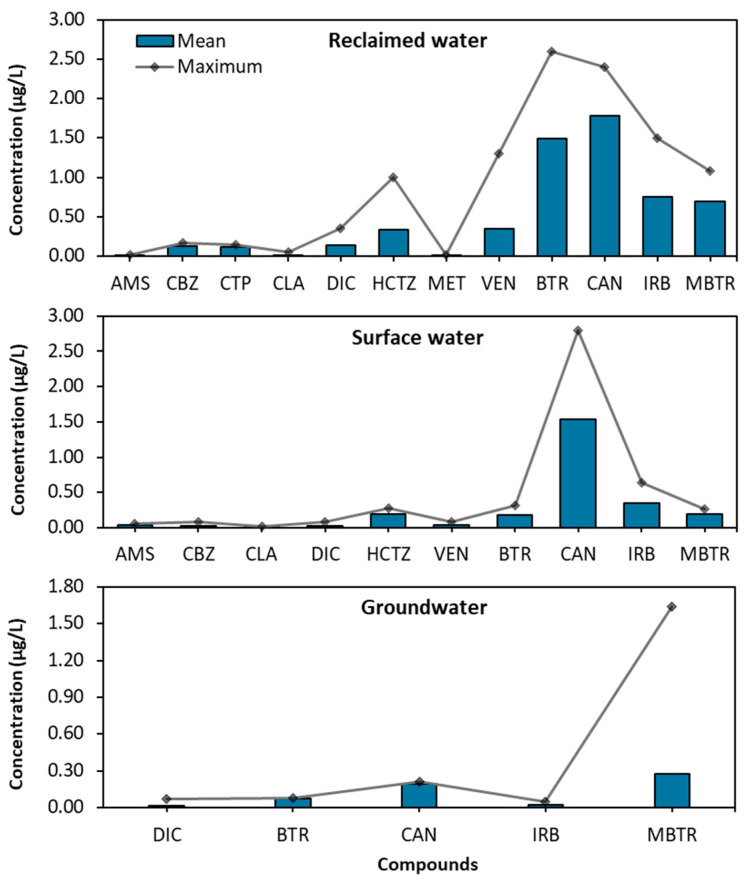
Mean and maximum concentrations of the twelve micropollutants in reclaimed water and surface water samples (n ≥ 6).

A comprehensive analysis of micropollutant concentrations was conducted on various water samples, including reclaimed water, surface water, and groundwater. Table 2 presents the minimum, maximum, and mean concentration values detected in the study, which were used to calculate risk quotients. Given the nature and heterogeneity of the samples, it is understandable to observe high variability in the data, which is reflected in some of the higher standard error values (Table 2). This variability could potentially be exacerbated by the detection limits or the presence of values below the limit of quantification (LOQ), which can influence the calculation of the error standard. These variations highlight the challenges associated with sampling from complex environments like natural water systems, where factors such as dilution, adsorption, and environmental interactions can contribute to the observed variability in some contaminant concentrations. Additionally, Figure 2 compares the mean and maximum concentrations of each compound across the different water sources. Compounds with concentrations below the limit of quantification were excluded from this comparative analysis.

The first category encompasses a variety of pharmaceuticals, including antidepressants, antipsychotics, antibiotics, anti-inflammatory drugs, diuretics, and beta-blockers. The detection of these substances across various environmental matrices is particularly concerning, as they contribute to antimicrobial resistance and pose significant risks to both environmental health and public safety. The comparison of maximum and mean concentrations presented in Figure 2 indicates that, for some compounds, the maximum values are very close to the averages. However, it is encouraging to note that the compounds with the highest maximum concentrations, such as benzotriazole, candesartan, and methylbenzotriazole, exhibit a significant divergence from the average values, with maximum concentrations at least 25% higher. This behavior suggests that despite the presence of elevated concentrations, their impact may be more manageable, allowing for better control of these contaminants in the environment.

In the first category of compounds, the highest average and maximum concentrations were generally observed in reclaimed water, followed by surface water and groundwater, with significant differences (*p* < 0.05) found among the three water types. However, Dunn’s post hoc test showed a significant difference (*p* < 0.05) only between reclaimed water and groundwater, while smaller differences (*p* < 0.1) were observed between surface water and groundwater. Venlafaxine exhibited the highest average concentration of 0.349 µg/L, with a peak of 1.300 µg/L in reclaimed water. Similarly, hydrochlorothiazide showed a notable average concentration of 0.337 µg/L, reaching a maximum of 1.000 µg/L in the same samples. Furthermore, diclofenac, citalopram, and carbamazepine were detected in nearly all samples, with concentrations around 0.150 µg/L in reclaimed water and below 0.080 µg/L in surface water and groundwater (Table 2). Although the surface water values were lower than those in reclaimed water, hydrochlorothiazide still showed the highest maximum concentration at 0.270 µg/L, with an average of 0.194 µg/L. The other compounds in category 1 in the surface water samples did not exceed 0.090 µg/L, with compounds like citalopram and metoprolol showing values below the LOQ. In groundwater, diclofenac was the only compound with notable concentrations, presenting a maximum of 0.070 µg/L and an average of 0.014 µg/L, while the remaining compounds were below the LOQ (Table 2). It is important to highlight that, despite some compounds displaying relatively high maximum concentrations, their average concentrations were consistently low and, in several cases, below the LOQ (see Supplementary Materials). This indicates that while peak concentrations can occasionally spike due to localized contamination events or fluctuations in environmental conditions, the overall presence of these compounds in the analyzed matrices remains relatively low on average. This pattern suggests that, although these compounds are detected at concerning levels intermittently, their sustained impact across time may be limited. This distinction between occasional spikes and consistent average levels is crucial for understanding both the short-term risks and the long-term management strategies needed to mitigate the presence of these contaminants in the environment.

The presence of pharmaceuticals, with a focus on human and ecological risk, has been extensively investigated in drinking water, providing a valuable reference point for comparing their concentrations across different environmental matrices. For example, Wang et al. (2023) [9] found that the maximum concentration of clarithromycin in drinking water did not exceed 0.002 µg/L. Similarly, Kondor et al. (2021) [18] evaluated diclofenac and carbamazepine in drinking water, reporting maximum concentrations of 0.004 µg/L and 0.080 µg/L, respectively. These levels are approximately five times lower than the maximum levels found in the various environmental samples evaluated in this current study. This disparity suggests differential exposure levels and highlights the importance of understanding how pharmaceuticals are distributed and transported within different environmental compartments. For instance, Martínez-Alcalá et al. (2021) [16] examined compounds such as diclofenac and carbamazepine in effluents from WWTPs within the Region of Murcia, finding that the maximum concentrations exceeded 0.100 µg/L. Although the analyzed effluents came from different treatment plants, these concentrations are consistent with those obtained in the present study, indicating similar contamination patterns and environmental behaviors. This connection reinforces the parallels between both studies. On the other hand, Singh and Suthar (2021) [34] found maximum concentrations of diclofenac below the detection limit and metoprolol at 0.024 µg/L in surface water samples from the Ganges River in India. In studies analyzing urban groundwater by Jurado et al. (2022) [35], the maximum reported concentrations of diclofenac and hydrochlorothiazide were significantly higher than those observed in this study, with values of 1.440 µg/L and 0.450 µg/L, respectively—exceeding the levels reported in this study by 2 to 3 times. Conversely, other compounds analyzed by these authors, such as carbamazepine and metoprolol, showed maximum concentrations of around 0.100 µg/L, which are higher than the values observed in this study, where concentrations were below 0.025 µg/L. This comparison highlights the variability in contaminant levels across different environments and underscores the need for context-specific assessments. This discrepancy underscores the differences in pharmaceuticals contamination levels across different water sources and emphasizes the potential risks associated with surface water and groundwater contamination. The presence of these contaminants, even at low levels, underscores the persistent and recalcitrant nature of pharmaceutical residues in the environment and their potential to pose risks over time [36].

In the second category, as with the first group of pharmaceuticals, the highest mean and maximum concentrations were predominantly found in reclaimed water (*p* < 0.05), followed by surface water, while groundwater exhibited significantly lower levels. This group comprises two antihypertensive pharmaceuticals, candesartan and irbesartan, along with two industrial chemicals, benzotriazole and methylbenzotriazole. Notably, these compounds showed elevated concentrations across all three water matrices, surpassing the levels observed in the first category. Candesartan and benzotriazole stood out with particularly high concentrations, with mean values of 1.783 µg/L and 1.498 µg/L, respectively. Their maximum concentrations were recorded at 2.400 µg/L for candesartan and 2.600 µg/L for benzotriazole, primarily in reclaimed water. In surface water, candesartan again emerged as the compound with the highest peak concentration, reaching 2.800 µg/L, indicating its potential mobility and persistence within aquatic ecosystems. Conversely, methylbenzotriazole displayed the highest maximum concentration in groundwater, peaking at 1.640 µg/L. This suggests that this industrial chemical may infiltrate deeper water reserves, raising concerns about long-term contamination of groundwater supplies. Although irbesartan exhibited lower concentrations overall, its levels remained significant in both reclaimed and surface waters, reflecting its continuous presence in these environments (Table 2).

When comparing these results with other studies, the widespread presence of candesartan in reclaimed and surface waters aligns with the findings of Castro et al. (2019) [37]. In their research, they conducted a comprehensive screening of environmental waters and treated effluents and reported similar levels of mean concentrations of candesartan residues. This consistent detection underscores a concerning trend regarding the accumulation of this pharmaceutical contaminant in aquatic ecosystems. Similarly, a study by Struk-Sokołowska et al. (2022) [38] and Wang et al. (2023) [39] detected high concentrations of benzotriazole in urban waters, with peak values reaching up to 2 µg/L in surface (rainwater) and treated water, closely aligning with the concentrations found in our study. The persistence and mobility of benzotriazole in aquatic environments have been well-documented, indicating that conventional wastewater treatment processes are often insufficient to fully remove this compound [40]. In contrast, the detection of methylbenzotriazole in the three water samples at concentrations as high as 1.400 µg/L suggests potential leaching through soil layers. This finding contrasts with the results of Zhang et al. (2023) [41], who reported lower, yet still concerning, levels of methylbenzotriazole in groundwater and surface water near industrial areas. This points to the compounds ability to penetrate deeper aquifers, raising concerns about long-term groundwater contamination and the challenges associated with remediating such pollution [40]. These elevated levels, detected across different environmental matrices, underscore the potential for these substances to enter the food chain, necessitating stricter regulatory measures and improved removal technologies in wastewater treatment plants to mitigate their impacts. According to the UWWTD regulation, methylbenzotriazole is evaluated as the sum of 4-methylbenzotriazole and 5-methylbenzotriazole, due to their shared environmental behavior and toxicological effects. Therefore, the Supplementary Materials includes the maximum and mean concentrations of each compound separately, offering a more comprehensive view of their environmental occurrence (Table S2, Supplementary Materials).

Lastly, it is important to highlight the differences in average and maximum concentrations among the various sample types, with slightly higher levels observed in reclaimed water. This variation can be attributed to the distinct origins of each sample type, despite all sampling points being influenced by discharged effluents from WWTPs. Specifically, the reclaimed water samples in this study originate directly from the effluent of the WWTP. Additionally, WWTP effluents typically discharge into surface water bodies where the water volume is significantly greater than that of the treated plant effluents. This larger volume of surface water can dilute emerging contaminants, thereby altering their concentrations compared to those found in WWTP effluents. Furthermore, as discussed by other researchers, emerging contaminants can undergo physical, chemical, and biological degradation processes within natural water matrices [42]. This is particularly relevant in the context of wastewater treatment, where processes like lagooning can significantly influence contaminant levels. For instance, Díaz-Gamboa et al. (2024) [43] reported a reduction in the concentration of certain emerging contaminants after the effluents from a WWTP underwent a lagooning stage. These degradation processes, influenced by various biotic and abiotic factors such as exposure to sunlight, temperature fluctuations, and microbial activity, can lead to notable changes in the occurrence and concentration of contaminants over time [42]. Understanding these dynamics is essential for assessing the long-term impacts of pharmaceutical and other emerging contaminants on water quality.

### 3.2. Human and Ecological Risk Quotients

The following results present the risk coefficients for the minimum, maximum, and mean concentrations. However, this study focuses on the ERQ and HRQ based on the maximum concentrations of the twelve indicators, as these offer a more accurate representation of the worst-case scenario. These concentrations are essential for assessing the water quality of effluents from WWTPs and understanding their potential impacts on public health and ecosystems. By calculating the HRQ and ERQ from these maximum concentrations, this research not only enhances our comprehension of the potential hazards posed by these substances but also provides a valuable framework that complements existing regulatory measures. Furthermore, this approach can inform water management policies in alignment with the UWWTD, ensuring a more integrated strategy for safeguarding environmental and human health.

#### 3.2.1. Human Risk Quotients

**Figure 3:**
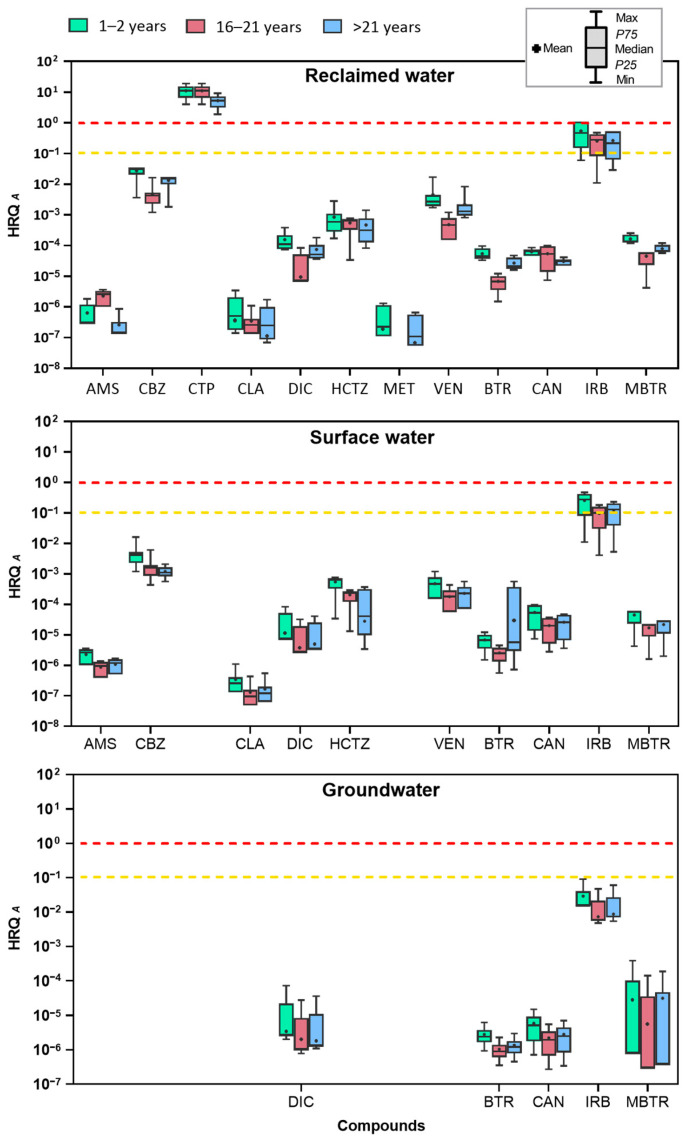
Human risk quotient for babies (aged 1 to 2 years), teenagers (aged 16 to 21 years), and adults (over 21 years) from emerging compounds found in reclaimed water, surface water, and groundwater samples, calculated using the primary methodology (Approach A). The box plots display the median, interquartile range, and the minimum and maximum values. The yellow dashed line indicates a risk quotient of 0.1 to 1 (moderate risk), and the red dashed line indicates a risk quotient above 1 (high risk).

To enhance the accuracy of human health risk assessments, this research expanded on prior studies that assessed the risks of emerging contaminants across different age groups [16,17,23]. Overall, the calculated HRQ (following Approach A) in this study ranged from 10^−7^ to 100 (Figure 3). Citalopram had the highest HRQ, followed by irbesartan. Although HRQs for babies were higher than those for teenagers and adults, most HRQ values were below 0.1, indicating no appreciable health concern. To provide a clearer visualization of the detected risk range, Figure 3 presents the full spectrum of HRQ values, from the minimum to the maximum detected, using boxplots. This article mainly discusses the results obtained with the primary approach (Approach A), while a second estimation (Approach B) is used to compare different calculation methods.

**Figure 4:**
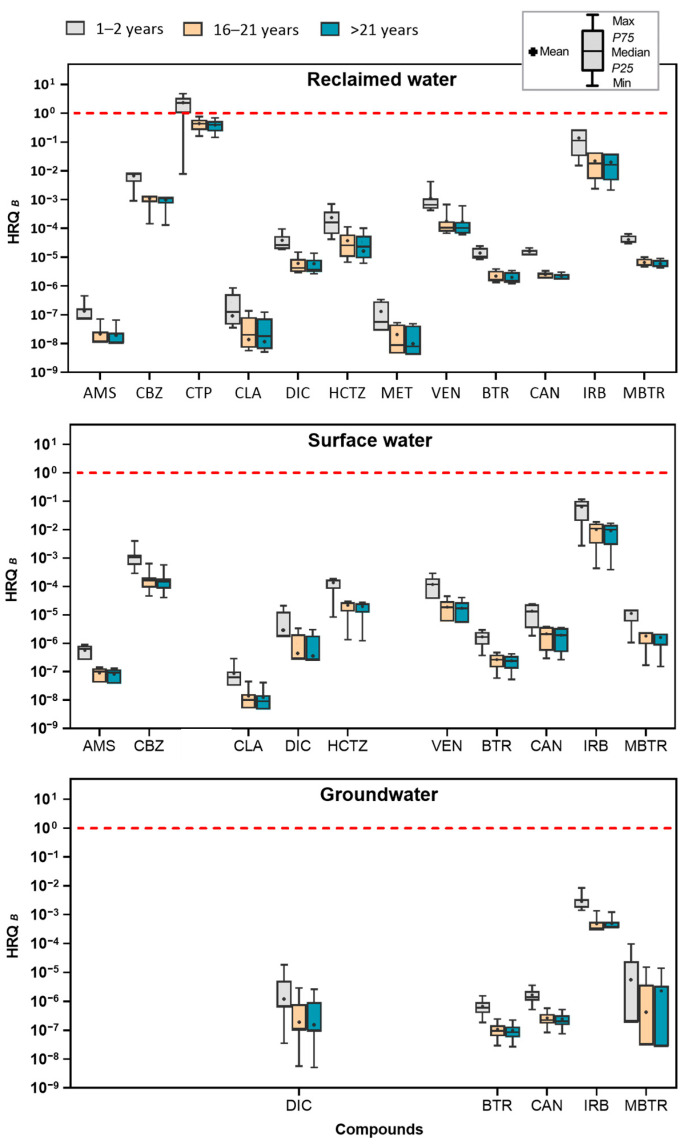
Human risk quotient for babies (aged 1 to 2 years), teenagers (aged 16 to 21 years), and adults (over 21 years) from emerging compounds found in reclaimed water, surface water, and groundwater samples, calculated using the alternative methodology (Approach B). The box plots display the median, interquartile range, and the minimum and maximum values. The red dashed line indicates a risk quotient above 1 (high risk).

When applying the alternative approach (Approach B), HRQ values ranged from 10^−^⁹ to 4, showing a reduced risk compared to Approach A (Table S3, Supplementary Materials). The only compound with elevated risk was citalopram when evaluated in babies in reclaimed water (Figure 4). These approaches enable a more comprehensive understanding of the risk levels across the entire dataset, emphasizing variations in exposure. To further explore the results of the calculated risk coefficients, the HRQ values for each group of compounds evaluated in this study will be discussed in greater detail.

The widespread use of pharmaceuticals poses threats not only to agriculture but also to the food chain through direct contamination and complex interactions within ecosystems. One area of concern is the detection of psychiatric pharmaceuticals like citalopram, which exhibited the highest HRQ values in reclaimed water, while in surface and groundwater, its HRQ was not evaluated as all measurements fell below the LOQ. Despite this, citalopram exceeded HRQ values of 1 in every case, posing a significant risk, particularly to babies (HRQ = 19.116), followed by adults (HRQ = 9.216) and teenagers (HRQ = 7.174) (Figure 3). When comparing these results with those obtained using the alternative approach (Approach B), the HRQ for citalopram was lower across all water types evaluated. Notably, in reclaimed water, the HRQ was lower, presenting high risk only for babies in reclaimed water (HRQ = 13.764). Unlike the primary approach, which showed high risks across all age groups, the alternative approach resulted in HRQ values below 1 for teenagers and adults, indicating a reduced risk in these groups (Figure 4). The psychotropic effects of citalopram, including potential alterations in mood, anxiety, and behavior, elevate the risk to mental health for exposed populations [44,45]. Babies are especially vulnerable, mainly due to their developing nervous systems, which may heighten their susceptibility to adverse effects from even minor exposure levels [46]. Consequently, the high HRQ values associated with citalopram call for urgent attention to the risks posed by these pharmaceutical residues in water, especially concerning vulnerable populations like babies.

In the case of antihypertensive pharmaceuticals, only irbesartan exhibited moderate to high human risk quotient levels across the three age groups evaluated (Figure 3). Babies were found to be the most susceptible, with an HRQ of 1.104 in reclaimed water samples, followed by adults (HRQ = 0.532) and teenagers (HRQ = 0.414), who both faced moderate risk (Table S3, Supplementary Materials). However, with the alternative approach (Approach B), irbesartan did not show any elevated risk across any of the water types evaluated in the different age groups, as the maximum risk value reached was lower, with an HRQ < 1 (Figure 4). The elevated risk for babies in the primary approach may be attributed to their lower body weight and higher relative water intake compared to older individuals [17]. Babies exposed to irbesartan may face significant health effects, including potential impacts on their developing cardiovascular systems. Since irbesartan is used to manage hypertension and other cardiovascular conditions, exposure during infancy could potentially disrupt normal cardiovascular development or contribute to developmental delays [18,47]. The moderate risk for older age groups emphasizes the importance of considering long-term exposure and cumulative effects, even when HRQ values are below the high-risk threshold. In the surface water samples, irbesartan also presented a moderate risk for all age groups, with babies having an HRQ of 0.471, followed by adults (HRQ = 0.227) and teenagers (HRQ = 0.177). Though lower than the values in reclaimed water, this still indicates a risk to humans, with ongoing exposure possibly compounding health impacts caused by other contaminants [48]. Conversely, no significant human risk was found in groundwater samples, suggesting that either irbesartan concentrations are too low to be hazardous or groundwater is not a primary exposure source. Despite the presence of other pharmaceuticals, such as candesartan and hydrochlorothiazide, their HRQ values were consistently below 0.1 in all water types, signifying a low human health risk from these compounds (Table S3, Supplementary Materials).

The frequent detection of certain pharmaceuticals in water samples underscores their widespread use and persistence, even after current wastewater treatment processes. This is particularly evident in the frequency percentages of occurrence for pharmaceuticals such as diclofenac, venlafaxine, and carbamazepine in the effluent samples of WWTP (reclaimed water) (Table 2). However, HRQ values of these compounds did not indicate a significant risk for any age groups, with values below 10^−2^ (Figure 3). These findings align with those of other studies. For instance, Martínez-Alcalá et al. (2021) [16] reported low HRQ values in different age groups for pharmaceuticals like diclofenac in WWTP effluents in the Region of Murcia. Similarly, Sharma et al. (2019) [17] found low HRQ values for pharmaceuticals, such as carbamazepine, in surface and groundwater samples. In contrast, Ramírez-Morales et al. (2020) [49] found high HRQ values for pharmaceuticals like diclofenac in WWTP effluents, with some values exceeding 100. Similarly, in the present study, significant differences in HRQ were observed between reclaimed water and groundwater (*p* < 0.05). Despite differences in value ranges, no significant difference was found between reclaimed water and surface water, while a significant difference was observed between surface water and groundwater (*p* < 0.1). These variations can be attributed to the matrix effect in treated wastewater or the diverse nature of environmental water, which can alter the chemical behavior of pharmaceuticals, leading to variations in detection levels and risk quotients across different water matrices. Factors such as the presence of organic matter, fluctuating pH levels, and interactions with other chemicals can influence the persistence and risk profiles of pharmaceuticals in treated wastewater compared to natural water bodies [50]. Moreover, while the risk varies across different studies due to the specific conditions of each study and the characteristics of the samples, the methodologies and approaches used to calculate risk and exposure references also contribute to the differences observed between studies.

Finally, pharmaceuticals with no detection and lower concentrations, such as amisulpride, clarithromycin, and metoprolol, did not present immediate significant risks; however, their persistence in the environment raises concerns about bioaccumulation in aquatic organisms, which could ultimately pose health risks to humans through the food chain [51,52]. Additionally, these substances may indirectly contribute to antibiotic resistance in environmental bacteria, further endangering human health by potentially reducing the effectiveness of antibiotics in medical treatments [9]. Continuous exposure to antibiotics, even at low concentrations, can drive the development of resistant bacterial strains, which may then be transmitted to human pathogens via contaminated water or food, making infections caused by these resistant bacteria more difficult to treat effectively [46,53]. The interpretation of HRQ values must also consider the limitations and uncertainties inherent in environmental risk assessments. Factors such as synergistic effects with other pollutants, seasonal variability, and changes in antibiotic usage patterns can influence the overall risk profile over time [46,54].

Regarding the HRQ values of the industrial chemicals benzotriazole and methylbenzotriazole, these were consistently below 10^−3^ in all water samples and for all age groups, indicating a low level of risk. Despite their widespread use in industrial applications, including corrosion inhibitors in antifreeze and de-icing fluids, the low HRQ values suggest that current concentrations in the analyzed water matrices pose minimal direct risk to human health. This is consistent with other studies, such as those by Wang et al. (2023) [39], which also found low risk associated with benzotriazole in waters. While methylbenzotriazole has been more extensively studied for its environmental impact, its assessment in terms of human health risks remains limited. Nonetheless, despite the immediate risk to human health appearing minimal, the persistence of these chemicals in aquatic environments remains a significant concern. Benzotriazole and its derivatives pose significant risks to human health, primarily due to their ability to disrupt the cell cycle. Studies have shown that these compounds exhibit mutagenic and carcinogenic effects in bacterial systems such as *Salmonella* and *Escherichia coli*, raising concerns about their potential as human carcinogens due to their genotoxic properties [38,55]. Additionally, benzotriazoles are highly resistant to biodegradation, raising concerns about their long-term accumulation in the environment, where they can remain structurally intact in water and sediments [56,57,58]. Over time, this persistence could lead to bioaccumulation in humans, particularly through the food chain, potentially increasing the risk of cancer and other serious health effects due to prolonged exposure [38,52,59,60,61]. Additionally, while current concentrations may not trigger significant health risks, seasonal fluctuations, increased industrial use, or changes in wastewater treatment efficiency could alter their risk profiles in the future. Therefore, continuous monitoring remains essential, particularly in areas where industrial activities are prevalent or where treated wastewater is reused for irrigation or other purposes. The potential for these compounds to interact with other contaminants, creating synergistic or additive effects, should also be considered in future risk assessments time [46,54].

In general, the interaction of pharmaceuticals with other pollutants and environmental stressors can exacerbate their impacts, amplifying the overall risk profile. Factors such as climate change, land use patterns, and wastewater treatment efficiency also influence the distribution and persistence of pharmaceuticals in the environment, necessitating adaptive management strategies to mitigate risks effectively [46,54]. Although ongoing monitoring of pharmaceutical residues in water bodies is essential for tracking trends and ensuring that levels remain within safe limits, certain conditions in the present study help mitigate immediate risks. When WWTP effluents are used for agricultural irrigation, additional barriers are in place that further reduce potential impacts. Additionally, neither the surface water nor groundwater in the study area falls within catchment areas designated for human drinking water, which reduces the likelihood of direct human exposure. In other studies, precipitation has been shown to play a key role in diluting surface and groundwater contaminants, thereby lowering the concentration of micropollutants. However, in the southeastern regions of Spain, low precipitation levels limit the potential for dilution in surface waters, which could otherwise help decrease the concentration of these pollutants. While the risk quotient values from this study indicate a low immediate risk to human health, improving wastewater treatment processes and managing agricultural runoff are still essential for addressing the longer-term impacts of pharmaceutical contamination. This integrated approach to managing emerging pollutants in water systems is vital to safeguard human health in the face of shifting environmental pressures.

Finally, the comparison of both human risk calculation approaches reveals important differences, highlighting the complexities inherent in risk assessment. These calculations are ultimately empirical estimations, as they rely on observed data but also incorporate theoretical modeling due to the lack of extensive in vivo studies. Although base data come from comprehensive studies, such as those conducted by the EPA, the methods used to calculate risk can introduce substantial variability across different approaches. Due to the lack of extensive studies and available data, values like the ADI and PNEC are often derived from empirical data combined with theoretical modeling. These models can inherently be subject to variability based on assumptions, data inputs, or modeling techniques. As a result, this variability can influence the interpretation of risk, potentially introducing uncertainty into the overall assessment. Nevertheless, the need to assess risks leads to the use of available values, even though they may originate from different derivations or methodologies.

In human risk assessment, both approaches assume a scenario of high and continuous exposure to the studied waters, which contributes to elevated risk values [17,32]. However, this assumption is somewhat disconnected from the current study’s reality, as the waters evaluated are not intended for human consumption, meaning exposure levels are much lower than those assumed in both approaches. Additionally, natural environments involve various physical, chemical, and biological processes that can attenuate and dilute contaminants, further reducing the exposure risk across water systems [43,62,63,64]. Despite these considerations, assessing risks remains crucial, particularly through worst-case scenarios. Such scenarios help monitor the potential risks posed by the presence of these compounds to human health, ensuring that potential threats are accounted for in environmental protection and policymaking.

#### 3.2.2. Ecological Risk Quotients

**Figure 5:**
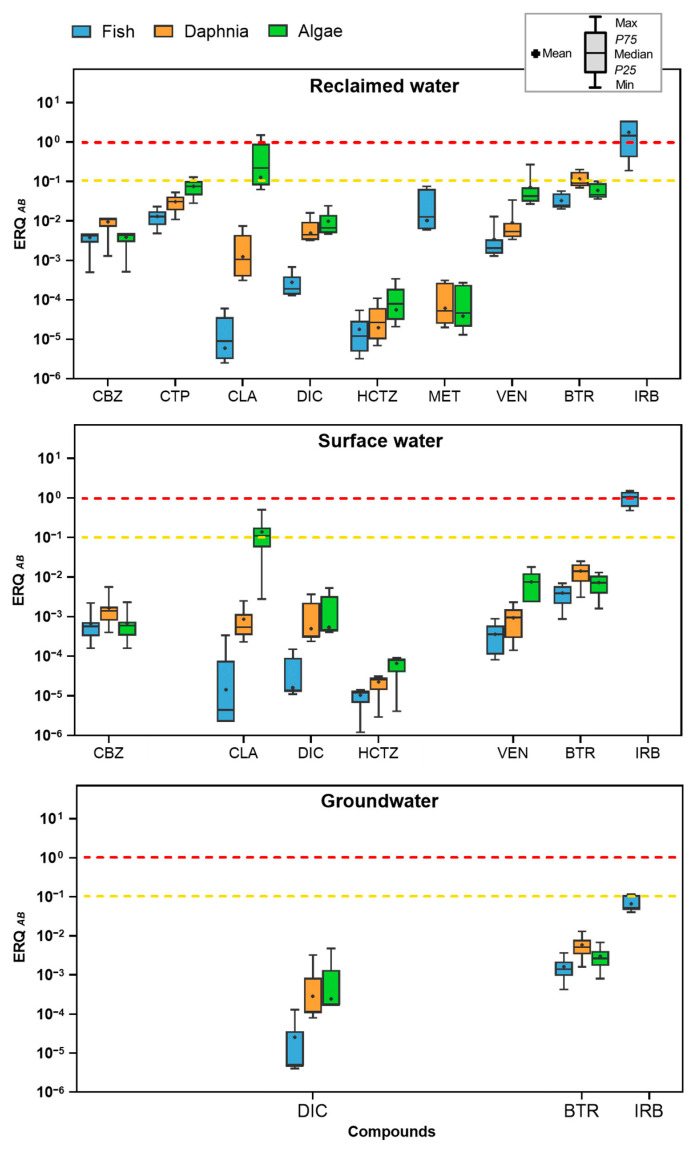
Ecological risk quotient (ERQ_AB_) for fish, daphnia, and algae from emerging compounds found in reclaimed water, surface water, and groundwater samples. The box plots display the median, interquartile range, and the minimum and maximum values. The yellow dashed line indicates a risk quotient of 0.1 to 1 (moderate risk), and the red dashed line indicates a risk quotient above 1 (high risk).

An important consideration in the ERQ analysis is that certain compounds, such as amisulpride, candesartan, irbesartan (for daphnia and algae), and methylbenzotriazole, had to be excluded due to the unavailability of key ecotoxicological data. The absence of PNEC values in the scientific databases, which are typically derived from parameters such as NOEC, LC_50_, or EC_50_, prevented their inclusion in the risk assessment for the studied species. According to Figure 5, the values of ERQ using the primary approach (Approach A) ranged from 10^−5^ to 10. According to Figure 5, the values of ERQ ranged from 10^−5^ to 10. The figure also shows the full spectrum of ERQ values, from the minimum to the maximum detected, using boxplots, further enhancing the understanding of the risk distribution across the dataset. Irbesartan exhibited the highest ERQ, followed closely by clarithromycin, both reaching high-risk levels. Among all the aquatic organisms evaluated (fish, daphnia, and algae), algae demonstrated the greatest susceptibility to these contaminants in most cases, with daphnia showing a similar trend. Furthermore, compounds such as citalopram and benzotriazole exhibited ERQ values ranging between 0.1 and 1, indicating a moderate ecological risk. In contrast, several substances in this study, including carbamazepine, diclofenac, and hydrochlorothiazide, had ERQ values below 0.1 in reclaimed water. A similar trend was observed in the surface and groundwater samples, where some compounds were consistently below the LOQ, leading to the exclusion of their ERQ evaluation. These low ERQ values suggest that the concentrations of these compounds, originating from WWTP effluents and other environmental samples, do not pose significant ecological risks when assessed individually.

For the ERQ estimation using the alternative approach (Approach B), the risk values remained consistent for the same compounds and organisms as in the primary approach (Approach A). This consistency arises because the alternative approach also includes the PNEC value in the ecotoxicological risk calculation, treating it as the GV. This underscores the importance of having reliable databases with baseline values to ensure accurate and consistent risk assessments in future analyses.

In the case of irbesartan, a high risk was observed for fish, particularly in reclaimed water (ERQ = 3.500) and surface water (ERQ = 1.493), while a moderate risk was noted in groundwater (ERQ = 0.107) (Table S4, Supplementary Materials). The presence of this pharmaceutical, even at low concentrations, can lead to chronic toxicity in fish, resulting in alterations in behavior, growth, and reproduction [47,48,65]. Furthermore, fish exposed to these substances may experience hormonal imbalances or disruptions in their endocrine systems, which can have long-term consequences for their populations and the overall health of aquatic ecosystems [66,67,68]. Despite an extensive literature search, PNEC values for irbesartan in daphnia and algae remain unreported, preventing their inclusion in the ERQ analysis and highlighting a significant gap in understanding the full ecological impact of this pharmaceutical. The absence of toxicity thresholds for key aquatic species limits the ability to fully characterize the potential environmental hazards posed by irbesartan and raises broader concerns about the implications of missing ecotoxicological data for other contaminants, particularly those considered indicators (including methylbenzotriazole) in surveillance regulations like the UWWTD. This data gap hinders comprehensive risk assessments, potentially leading to an underestimation of environmental impacts. However, despite these limitations, this study covers the majority of the 12 compounds identified in the UWWTD, providing a solid foundation for assessing the ecological risks associated with these key indicators. Future research should prioritize the development of predictive models that integrate existing data and extrapolate the risks for compounds lacking empirical PNEC values, while also conducting experimental studies to directly assess the toxicity of these compounds in key species. This would enhance the overall ecological risk assessment, ensuring a more robust evaluation of pharmaceutical contaminants and other emerging pollutants, and ultimately informing regulatory frameworks for environmental protection.

In addition to irbesartan, clarithromycin also posed a high risk, particularly in reclaimed water (ERQ = 1.500), and a moderate risk in surface water (ERQ = 0.500), primarily impacting algae (Table S4, Supplementary Materials). Despite the absence of significant concentration peaks for this antibiotic, its elevated risk can be attributed to the low PNEC values for algae, which make this species particularly sensitive to clarithromycin exposure. The impacts of antibiotics on algal populations can initiate cascading effects throughout the aquatic ecosystem, including the disruption of photosynthesis [12]. Since algae are fundamental to aquatic food webs, their disruption can negatively affect higher organisms such as fish and invertebrates, ultimately altering the balance and functioning of the entire ecosystem [12,69]. This disruption can lead to reduced biodiversity, altered nutrient cycling, and changes in ecosystem dynamics. These findings are consistent with previous studies that have highlighted similar environmental risks associated with antibiotic residues. Research in Spain [54,70,71] and other countries [49,72] has documented the pervasive issue of antibiotics contamination and its ecological impacts. The widespread presence of antibiotics in aquatic environments not only threatens the health of individual species but also has broader implications for ecosystem stability and resilience.

One compound that demonstrated moderate risk is citalopram, particularly affecting algae due to its low PNEC value for this species (Figure 5). Similarly, another psychoactive compound, venlafaxine, exhibited moderate toxicity to algae in reclaimed water, with an ERQ of 0.271. This raises growing concerns about psychoactive drugs, as they are designed to alter human behavior and mental states and may also have side effects that could influence fitness-related traits in free-living animals [45]. The broader implications of these medications in the environment are concerning; they interact with neurotransmitter systems and can affect the nervous systems of non-target organisms. Such interactions may lead to unintended and possibly detrimental effects on their behavior, physiology, and fitness-related traits [26,44]. For instance, changes in feeding, mating, and predator avoidance behaviors could occur, which may reduce the survival and reproductive success of affected animals [44,45,73]. These impacts might not be immediately evident but could lead to long-term changes in population dynamics and ecosystem structure. Therefore, while the immediate risk posed by most psychiatric pharmaceuticals might be low, the potential for more insidious and long-term effects remains a significant concern for environmental scientists [73,74,75].

On the other hand, benzotriazole, as an industrial chemical, also posed a moderate risk in reclaimed water, primarily due to the high maximum concentration detected in these samples. This elevated risk was particularly significant for sensitive aquatic species such as daphnia and algae, which can experience adverse effects like inhibited growth, reproduction, and photosynthesis [38]. However, in the surface and groundwater samples, benzotriazole posed a lower ecological risk, with ERQ values indicating minimal concern for these environments (Figure 5). Nevertheless, chronic exposure to even low concentrations of industrial chemicals like benzotriazole could still contribute to long-term imbalances in ecosystems, affecting food chains and biodiversity over time [38,41]. In the case of methylbenzotriazole, another industrial chemical of concern, its ecological risk could not be fully assessed due to limited data. While the UWWTD treats these two compounds collectively rather than individually, this study evaluated the ecological risks of each substance separately. The results showed that their impact on the studied waters is minimal, with risk values consistently falling below 10^−^² for all species (Table S4, Supplementary Materials). This is particularly noteworthy given that both compounds are known for their persistence in aquatic environments and are frequently detected in surface waters and wastewater effluents, especially near industrial areas where they are used as corrosion inhibitors. Their resistance to degradation processes, such as photolysis and biodegradation, allows them to persist and accumulate in these ecosystems, raising significant concerns about their potential ecological impacts [38,40].

Finally, compounds with low-risk values (ERQ < 10^−2^), such as diclofenac, despite their high frequency of occurrence, exhibited low maximum concentrations and risk levels for all species (Figure 5). Martínez-Alcalá et al. (2021) [16] corroborated this by finding that diclofenac concentrations in WWTP effluent samples did not pose any significant risk to the evaluated species. Nevertheless, it is essential to note that diclofenac has been linked to adverse effects in fish, including alterations in liver function and changes in reproductive behavior [76]. This widely used anti-inflammatory drug is frequently detected in various aquatic environments due to extensive consumption and only partial elimination during wastewater treatment processes. Its persistence can lead to chronic exposure, posing risks to non-target organisms [45,69]. In addition to diclofenac, other pharmaceuticals, such as carbamazepine and hydrochlorothiazide, also showed low ERQ values, particularly in surface water. In groundwater, however, the ERQ values could not be evaluated because their concentrations were below the LOQ. These findings align with those reported by Sharma et al. (2019) [17], who indicated a similarly low ecological risk for these substances. However, it is important to acknowledge that even at lower concentrations, these compounds can still represent a potential risk for chronic exposure in both aquatic and terrestrial environments [77,78,79], thereby warranting ongoing monitoring and research. Moreover, both the average and maximum concentrations of the studied compounds exhibited significant variability, ranging from very low to very high levels (Table 2). This variability suggests differential degradation rates or excretion patterns, which likely influence their environmental impact and potential for bioaccumulation [36]. For instance, compounds that degrade slowly or are excreted in large quantities may persist in the environment longer, increasing their ecological footprint. Bioaccumulation can lead to higher concentrations of these substances in the tissues of aquatic organisms, potentially causing toxic effects even if environmental concentrations are low [36,52,80,81]. In fish, chronic exposure to certain pharmaceuticals has been associated with developmental delays, altered metabolic rates, and changes in growth patterns, leading to reduced body size or asymmetrical growth [79,82,83]. Additionally, bioaccumulated contaminants can interfere with endocrine signaling, disrupting reproductive cycles and reducing fertility, which may compromise population stability in affected ecosystems [15,84]

Generally, the environmental risk assessment of pharmaceutical is fraught with uncertainties due to limited knowledge about their fate in waste and the environment, their absorption, metabolism, and excretion in wildlife, and their interactions with non-target species [45]. These compounds are particularly potent, and their continuous, low-level release into the environment is likely to cause chronic effects. Some pharmaceuticals have modes of action that can be particularly harmful, such as cytostatic effects or endocrine modulation. Additionally, these substances can have both direct and indirect effects, including through the food chain, and may interact with other stressors in additive or synergistic “cocktail” effects [45,85]. Understanding these dynamics is crucial for developing more effective environmental protection measures and drug disposal guidelines. The variability in concentration profiles underscores the need to enhance analytical techniques and further investigate the degradation pathways and environmental fate of these substances.

On the other hand, like human risk assessment, the empirical and theoretical approaches and methodologies used in ecological risk evaluation often assume high exposure scenarios based on worst-case conditions. These methods do not account for the various physical, chemical, and biological processes in environmental waters that could lead to the degradation of contaminants, ultimately reducing the overall risk. Therefore, it is important to evaluate the environment from both local and global perspectives when applying these methodologies, ensuring a more comprehensive understanding of the potential risks that may arise.

In the context of the UWWTD, it is essential not only to assess the removal of the 12 indicators as established by the regulations but also to evaluate whether the resulting concentrations could pose potential risks to human health. The specific conditions of the WWTP and the environmental matrix can significantly influence the effectiveness of contaminant removal. Consequently, the regulations may not fully capture the risks associated with exposure to these substances, especially when final concentrations still exceed levels considered safe. Therefore, assessing potential human and ecological risks is crucial for understanding how contaminants interact with the environment and affect public health, going beyond removal percentages. A comprehensive evaluation of both contaminant removal and associated risks is vital for effective environmental management and ensuring the safety of water used in irrigation and other applications.

## 4. Conclusions

It is important to note that the waters studied in this research, particularly the reclaimed water, are not intended for human consumption under any circumstances. However, potential risks associated with these waters were monitored in the hypothetical scenario of high human exposure or consumption. This study has identified certain pharmaceuticals, including citalopram, irbesartan (HRQ > 1 in babies), and clarithromycin (ERQ > 1 in algae), as well as industrial chemicals like benzotriazole (ERQ > 0.1 in Daphnia), as posing significant risks to human and ecological health, exceeding the moderate and high-risk thresholds. In contrast, pharmaceuticals such as carbamazepine, hydrochlorothiazide, metoprolol, and diclofenac were found to present a lower risk in the studied waters (Approach A). These findings are based solely on the evaluation of maximum concentrations, which serve as a reference point or worst-case scenario for assessing potential risks. In the case of human risk assessment specifically, the application of a second risk assessment approach (Approach B) revealed that fewer cases exceeded the high-risk threshold compared to the primary approach (Approach A). Specifically, compounds such as irbesartan and clarithromycin exhibited lower risk levels in most cases, with their values falling below the risk threshold of 1, thus highlighting the contrasting outcomes between the two approaches.

This discrepancy underscores the need to approach risk assessment from both a specific and a broader, global perspective. Although the waters studied in the present research are not designated for human consumption, both approaches rely on reference values derived from scenarios assuming human consumption. These values, such as ADI and DWEL for human risk assessment, and PNEC for ecological risk assessment, are based on different methodologies and can lead to significant variations and discrepancies in the reported data. Furthermore, risk assessment models often assume high exposure scenarios, and the choice of empirical or theoretical models further influences the calculated risks, contributing to discrepancies across different studies and scenarios. It is important to consider these worst-case exposure scenarios to enable effective monitoring and follow-up of potential risks, particularly in the hypothetical case of high human exposure.

A notable finding of this research is that the ecological risks associated with these contaminants were significantly higher than those related to human health. Although human health risks reached very high values compared to ecological risks, the latter encompassed a greater number of compounds posing significant risk. This discrepancy may stem from the higher acceptable daily intake values established for humans or the greater susceptibility of aquatic organisms to these pollutants.

The maximum concentrations of these compounds emerged as a crucial factor in determining the extent of the associated risks. Notably, effluent samples showed the highest maximum concentrations among all evaluated samples, reaching up to 2600 µg/L for industrial chemicals such as benzotriazole and 2400 µg/L for pharmaceuticals such as candesartan. In contrast, surface waters exhibited intermediate concentration levels, while groundwater consistently showed the lowest average and maximum concentrations, often falling below the limit of quantification for most of the evaluated compounds.

From a policy perspective, these findings underscore the urgent need for stronger regulations on the discharge and treatment of pharmaceuticals and other emerging contaminants. Regulatory frameworks should evolve to incorporate more rigorous monitoring protocols and promote advanced wastewater treatment technologies capable of effectively removing these compounds. Additionally, it is crucial to establish clear guidelines for sustainable water reuse, ensuring a balance between agricultural needs and environmental protection. Furthermore, policymakers should mandate environmental risk assessments as part of the approval process for new pharmaceuticals, ensuring that their long-term ecological effects are carefully evaluated before widespread use.

In terms of future research, this study highlights the need for long-term monitoring programs to gain a more comprehensive understanding of contaminant persistence and transformation in aquatic environments. Further investigations should focus on the impact of chronic low-dose exposure on both ecosystems and human health. Additionally, research should explore the combined effects of multiple contaminants, as real-world exposure scenarios typically involve complex mixtures rather than isolated compounds. Ultimately, the development of environmentally safer pharmaceuticals, designed to minimize persistence and toxicity, represents a crucial research avenue to help mitigate long-term contamination risks.

Finally, addressing pharmaceutical pollution effectively requires interdisciplinary collaboration between scientists, policymakers, and industry stakeholders. Eco-friendly drug design, improved waste management strategies, and public awareness campaigns are all crucial components of a holistic approach to mitigating the risks associated with these emerging contaminants. Ensuring water safety in regions with high levels of wastewater reuse, such as the Murcia Region, will be fundamental to protecting both human health and ecosystem integrity in the face of increasing contamination by pharmaceuticals and industrial chemicals.

## Figures and Tables

**Table 1:** Chemical Abstract Service (CAS) registry number, acceptable daily intake (ADI), values of DWEL for different ages and PNEC for different reference organisms of the emerging compounds studied.

Category	Compound	CAS N°	ADI µg/(kg bw Day)	DWEL (µg/L)			PNEC (µg/L)		
				1–2 Years	16–21 Years	>21 Years	Fish	Daphnia	Algae
Category 1	Amisulpride ^a^	71675-85-9	1020	14,436	38,466	29,943	-	-	-
	Carbamazepine ^b^	298-46-4	0.340	4.812	13	10	35	14	34
	Citalopram ^de^	59729-33-8	0.001	0.011	0.029	0.023	9.136	3.900	1.598
	Clarithromycin ^a^	81103-11-9	1230	17,408	46,385	36,108	1000	8.16	0.040
	Diclofenac ^b^	15307-86-5	67	948	2527	1967	530	22	15
	Hydrochlorothiazide ^b^	58-93-5	25	354	943	734	18,680	8740	2920
	Metoprolol ^a^	51384-51-1	1050	14,861	39,597	30,824	0.267	64	73
	Venlafaxine ^ef^	93413-69-5	5.401	76	204	159	100	38	4.800
Category 2	Benzotriazole ^a^	95-14-7	1900	26,891	71,652	55,776	46	13	25
	Candesartan ^a^	139481-59-7	2000	28,306	75,423	58,712	-	-	-
	Irbesartan ^c^	138402-11-6	0.096	1.359	3.620	2.818	0.429	-	-
	Methylbenzotriazole ^a^	29385-43-1	300	4245	11,313	8806	-	-	-
	4-Methylbenzotriazole ^gh^	29878-31-7	30	419	1116	869	197	109	73
	5-Methylbenzotriazole ^ag^	136-85-6	262	3708	9880	7691	197	94	73

The reference values for ADI, DWEL, and PNEC calculations were obtained from the following sources: ^a^ The United States Environmental Protection Agency (EPA) website (https://www.epa.gov/), accessed on 16 June 2024; ^b^ Sharma et al. (2019) [17]; ^c^ Charoo et al. (2019) [24]; ^d^ Silva et al. (2017) [25]; ^e^ Bouzas-Monroy et al. (2022) [26]; ^f^ Liu et al. (2019) [27]; ^g^ Zhao et al. (2024) [28]; ^h^ Yang et al. (2022) [29].

**Table 2:** Frequency of occurrence (FRO), range (minimum and maximum), mean concentrations, and standard error (SE) of the twelve micropollutants detected in reclaimed water, surface water, and groundwater samples.

Compound	Reclaimed Water				Surface Water				Groundwater			
	FRO (%)	Min–Max (µg/L)	Mean (SD) (µg/L)	SE	FRO (%)	Min–Max (µg/L)	Mean (SD) (µg/L)	SE	FRO (%)	Min–Max (µg/L)	Mean (SD) (µg/L)	SE
Amisulpride (AMP)	17	0.004–0.026	0.008 (0.008)	0.002	60	0.015–0.052	0.033 (0.015)	0.005	ND	<LOQ	<LOQ	NA
Carbamazepine (CBZ)	88	0.018–0.170	0.132 (0.051)	0.015	50	0.006–0.078	0.023 (0.020)	0.007	ND	<LOQ	<LOQ	NA
Citalopram (CTP)	83	0.044–0.150	0.120 (0.053)	0.015	ND	<LOQ	<LOQ	NA	ND	<LOQ	<LOQ	NA
Clarithromycin (CLA)	25	0.003–0.060	0.019 (0.020)	0.006	22	0.002–0.020	0.006 (0.005)	0.002	ND	<LOQ	<LOQ	NA
Diclofenac (DIC)	88	0.070–0.360	0.147 (0.095)	0.027	33	0.007–0.080	0.024 (0.022)	0.008	13	0.003–0.070	0.014 (0.023)	0.006
Hydrochlorothiazide (HCTZ)	100	0.060–1.000	0.337 (0.316)	0.091	60	0.012–0.270	0.194 (0.093)	0.033	ND	<LOQ	<LOQ	NA
Metoprolol (MET)	17	0.002–0.020	0.008 (0.008)	0.002	ND	<LOQ	<LOQ	NA	ND	<LOQ	<LOQ	NA
Venlafaxine (VEN)	88	0.130–1.300	0.349 (0.367)	0.106	56	0.011–0.088	0.036 (0.027)	0.010	ND	<LOQ	<LOQ	NA
Benzotriazole (BTR)	100	0.900–2.600	1.498 (0.654)	0.189	80	0.040–0.320	0.182 (0.091)	0.032	83	0.020–0.080	0.074 (0.045)	0.016
Candesartan (CAN)	100	1.300–2.400	1.783 (0.398)	0.141	80	0.210–2.800	1.534 (1.043)	0.369	83	0.060–0.210	0.191 (0.110)	0.039
Irbesartan (IRB)	71	0.082–1.500	0.753 (0.563)	0.230	60	0.015–0.640	0.348 (0.220)	0.090	17	0.021–0.046	0.025 (0.009)	0.004
Methylbenzotriazole (MBTR)	100	0.501–1.080	0.702 (0.218)	0.063	100	0.018–0.260	0.192 (0.092)	0.032	17	0.003–1.640	0.276 (0.601)	0.193

LOQ: Limit of Quantification. ND: Not Detected. NA: Not Applicable. SD: Standard Deviation. FRO: did not consider values below quantifiable levels.

## Data Availability

The data presented in this study are available on request from the corresponding author.

## Supplementary Materials

The following supporting information can be downloaded at https://www.mdpi.com/article/10.3390/toxics13040275/s1. Table S1: Description of Sampling Points for Micropollutant Analysis in the Region of Murcia, Spain; Table S2: Data for methylbenzotriazole and its derivatives, 4-methylbenzotriazole and 5-methylbenzotriazole, from samples of reclaimed water, surface water, and groundwater; Table S3: Acceptable Daily Intake (ADI), Drinking Water Equivalent Level (DWEL) values, and Human Risk Quotient (HRQ) calculated for three different age groups of the twelve studied micropollutants, based on their maximum concentrations; Table S4: Predicted No-Effect Concentration (PNEC) and Environmental Risk Quotient (ERQ) for different reference organisms of the twelve studied micropollutants, based on their maximum concentrations. The data include the limit of quantification (LQ).

## Abbreviations

The following abbreviations are used in this manuscript:

ADI	Acceptable Daily Intake
AMS	Amisulpride
BTR	Benzotriazole
CAN	Candesartan
CAS	Chemical Abstract Service
CBZ	Carbamazepine
CIT	Citalopram
CLR	Clarithromycin
DIC	Diclofenac
DWEL	Drinking Water Equivalent Level
EC50	Effective Concentration for 50% of the population
ECHA	European Chemicals Agency
ERQ	Ecological Risk Quotient
GV	Guideline Value
HCTZ	Hydrochlorothiazide
HRQ	Human Risk Quotient
IARC	International Agency for Research on Cancer
IRB	Irbesartan
LC50	Lethal Concentration for 50% of the population
LOQ	Limit of Quantification
MBTR	Methylbenzotriazole
MEC	Measured Environmental Concentration
MET	Metoprolol
NOEC	No Observed Effect Concentration
PNEC	Predicted No-Effect Concentration
TMP	Trimethoprim
UWWTD	Urban Wastewater Treatment Directive
VEN	Venlafaxine
WWTP	Wastewater Treatment Plant
